# Effect of Biopolymer Dip-Coating Pretreatments as a Non-Thermal Green Technology on Physicochemical Characteristics, Drying, and Rehydration Kinetics of Santa Maria Pears

**DOI:** 10.3390/foods12132466

**Published:** 2023-06-23

**Authors:** Nasim Kian-Pour

**Affiliations:** Gastronomy and Culinary Arts Department, Faculty of Fine Arts, Istanbul Aydin University, 34295 Istanbul, Turkey; nasimkianpour@aydin.edu.tr

**Keywords:** non-thermal processing, biopolymer, dip-coating, green technology, rehydration kinetics, pear

## Abstract

This research was conducted to determine the influences of biopolymer dip-coating pretreatments as a non-thermal green technology on the drying behavior, retention of bioactive compounds, and quality properties of pears. The fresh pears were washed, peeled, and diced into cubes of 5 × 5 mm with a 2 mm thickness and were dipped into 0.3% (*w*/*v*) solutions of sodium alginate (SA), pectin (PC), xanthan gum (XG), Arabic gum (AG), and gelatin (GE) before hot air drying (70 °C, 2.0 m/s). The weight loss of samples during drying was recorded online, and the moisture ratio (MR) and drying rate were plotted against drying time. Biopolymers significantly decreased the drying time (maximum 33.33% by SA) compared with uncoated samples except for XG. Moisture diffusion coefficients were determined according to Fick’s second law of diffusion by plotting LnMR against drying time, and a linear regression analysis was applied to the data for the determination of moisture diffusion coefficients which ranged from 2.332 to 3.256 × 10^−9^ m^2^/s. The molecular transport of momentum, heat, and mass were determined from Newton’s law of viscosity, Fourier’s law, and Fick’s law, respectively. The results indicated that the friction drag force, convective heat, and mass transfer coefficients were 6.104 × 10^−6^ N, 76.55 W/m^2^·K, and 0.0636 m/s, respectively. Mathematical modeling showed the suitability of the Midilli and Kucuk and the Peleg models for the prediction of drying and rehydration processes, respectively. Thermal conductivity, specific heat, and density of coated samples ranged from 0.559–0.579 (W/m·K), 3735–3859 (J/kg·K), and 850.90–883.26 (Kg/m^3^), respectively. The porosity was reduced due to the penetration of biopolymers into the cellular matrix of samples. The highest total polyphenol content and antioxidant activity belonged to the AG samples. The biopolymers covering the surface of samples produced a protection layer against the loss of bioactive compounds. Biopolymers can be successfully used as a non-thermal green process for improving the drying and quality characteristics of pears at the industrial level.

## 1. Introduction

Biopolymers are a leading class of macromolecules with distinctive characteristics. They are widely used in food, cosmetic, medicine, environmental protection, analytical chemistry, and materials engineering. Biopolymers can be categorized as either natural or synthetic according to their origin [1]. Natural biopolymers such as hydrocolloids are a large class of high-molecular-weight, long-chain hydrophilic macromolecules that contain different functional groups [2]. According to their sources, chemical structures, and ionic attributes, they exhibit complete or partial solubility by forming hydrogen bonds with water [2,3]. They can change the properties of the aqueous solution by forming a gel, thickening the solution, or stabilizing the emulsion [3,4]. Their solubility in the aqueous solution in food and gastrointestinal liquids is an important factor in their functionality [4]. Food biopolymers such as carbohydrates and proteins are the most important biopolymers, considered clean-label food additives [5]. Many biopolymers can be used as dietary fiber, acting as health promoters in reducing the risk of many health problems such as cardiovascular disease, diabetes, and colon cancer [3,6]. In addition, they can be utilized as an edible coating on the surface of products to preserve the quality properties of food [7,8]. Moreover, the ability of biopolymers to carry antioxidants and bioactive compounds improves their protective properties by limiting the loss of biologically active nutrients during different food processes, such as drying [9]. Biopolymer coating impacts the drying efficiency and energy consumption of dryers by influencing heat and mass transfer during drying [10]. For instance, the strong polarity of proteins and polysaccharides biopolymers gives them low moisture barrier properties. These polymers also have high water solubility coefficients, resulting in high rates of water vapor penetration and, as a result, low resistance to mass transfer during drying. Consequently, they can improve the drying characteristics of food [11]. Furthermore, it can be considered a non-thermal pretreatment and a potential substitute for thermal pretreatments (such as hot-water blanching, microwave, and infrared treatment) before drying to decrease the drying time and improve the quality properties of samples [6]. Therefore, biopolymer coating as a green technology shows a very important effect on both quality properties of food (such as color, nutrient retention, rehydration, and microstructure) and environmental aspects (by decreasing the thermal processes, resulting in reduced global warming and CO_2_ footprint) [9].

Pears (*Pyrus* spp.) are one of the most widely consumed fruits in the world due to their wonderful taste and health benefits [12]. Pears contain various bioactive compounds contributing to human health due to their protective properties against many health problems, including inflammation, cancer, diabetes, and cardiovascular disease [13,14]. However, due to their high moisture content, pears are sensitive to microbial spoilage, softening, and enzymatic browning [12]. Therefore, convective drying, considered the most common industrial drying method, can be used to protect pears from unwanted deterioration. Dried pears in the form of slices, pieces, or powders can be used in sauces formulation, dried salad seasoning, fruit teas, drinks, cocktails, dried fruit snacks, cake mixes, bakery, and confectionery products. However, not only does long drying time in convective drying harm the quality properties of fruits, but it also increases energy consumption and global warming; thus, drying must happen quickly [15]. Convective drying is a highly energy-intensive process, using thermal energy for drying food products. However, special attention needs to be paid to eliminating the drawbacks of deficiency and the rising cost of various fuels as well as saving energy in industrial operations [16]. Nowadays, the international effort aims to transition to green technologies to reduce the changes associated with thermal energy consumption, global climate change, carbon footprint, environmental contamination, and air pollution.

Non-thermal prior-drying pretreatment, such as biopolymer coating, is an alternative method to accelerate the drying operation, decrease energy consumption, and improve the quality of dried products [10]. Morodi, Kaseke, and Fawole (2022) [9] reported that dip coating of red raspberries in AG solution (3% to 10% *w*/*v*) before oven drying at 60 °C improved hardness, ascorbic acid content and total phenolic content (TPC) of samples compared with uncoated samples. The authors demonstrated that pretreatment at 3% and 5% AG solutions could improve the antioxidant properties and physicochemical characteristics of red raspberries. Jansrimanee and Lertworasirikul (2022) [17] confirmed that a combination of ultrasound and SA coating decreased the osmotic dehydration time of pumpkin cubes. Rodriguez, Soteras, and Campanone (2021) [18] reported that the pear cubes coated with SA preserved the phenolic compounds (31.4%) better than uncoated (26.04%) and PC (20.77%) samples. However, transport properties such as momentum transfer, heat transfer coefficient, mass transfer coefficient, thermophysical properties, chemical bonds, crystalline structure, and thermal properties have rarely been investigated by the authors.

Many dried products are rehydrated before usage. Rehydration is a complex process in which dried food is immersed in a liquid (generally water). The rehydration process contains three stages: water absorption, swelling of the sample, and leaching of soluble material from the sample to liquid [19]. The microstructural integrity of foods during drying had a great impact on the rehydration characteristics of samples [20]. Generally, water absorption takes place quickly at the beginning of the rehydration process, while the rate of water absorption of samples gradually decreases with the progress of rehydration due to reaching the equilibrium moisture content [21]. Maintenance of the integrity of pores, capillaries, and intact cell walls during drying has an important effect on the transport of water during rehydration [22]. The rehydration process can be used as a quality criterion representing the microstructural and chemical changes during drying. The knowledge of the rehydration behavior of dehydrated food is extremely important for enhancing the quality of dried and rehydrated foodstuff [19]. Various authors studied the rehydration properties of food products such as pineapple [23], squid fillets [24], mango [20], dried apple [19], and scallop adductors [10].

However, the studies about dried pears have been restricted to hot air-dried uncoated pears or osmotic dried pears coated with coating materials. To the best of our knowledge, the dip-coating of fresh Santa Maria pears in SA, AG, PC, XG, and GE before hot air drying has not been studied. Therefore, the main aim of this investigation was to show the importance of using biopolymers as a non-thermal green technology in improving the drying process. This study was conducted to evaluate the effect of the biopolymer dip-coating pretreatment of fresh cubes (5 × 5 × 2 mm) of Santa Maria pears on the drying characteristics, rehydration kinetics, total polyphenol content, antioxidant activity, mathematical modeling of drying and rehydration, transport properties, microstructure, thermal properties, chemical bonds, and crystalline structure of dried pear.

## 2. Materials and Methods

### 2.1. Materials

Type B gelatin from bovine skin (EC. No.: 232-554-6, Sigma-Aldrich, St. Louis, MO, USA), SA (No.: 9005-38-3, Sigma-Aldrich, St. Louis, MO, USA), low methoxyl PC (EC. No.: 232-553-0, Sigma-Aldrich, St. Louis, MO, USA), XG (EC. No.: 234-394-2, Sigma-Aldrich, St. Louis, MO, USA) and AG (EC. No.: 232-519-5, Sigma-Aldrich, St. Louis, MO, USA), and calcium chloride (EC. No.: 232-140-8, Sigma-Aldrich, St. Louis, MO, USA) were used in this study.

### 2.2. Sample Preparation

**Figure 1:**
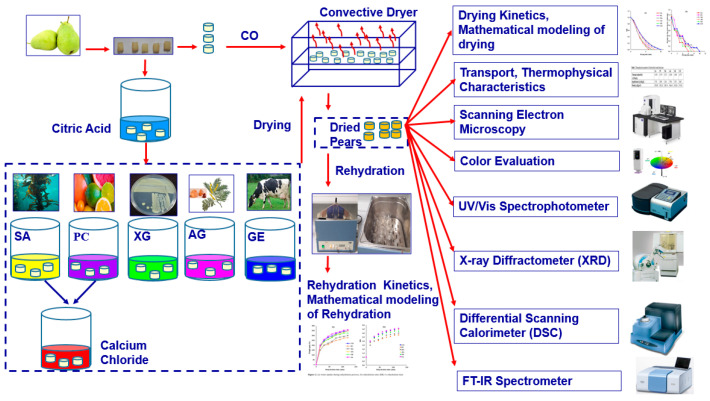
Schematic diagram of biopolymer-dip-coated dried pears.

The schematic diagram of the whole process is shown in Figure 1. Fresh Santa Maria pears in the commercial ripening stage at medium size (diameter of 70 mm) were provided from the local market (Istanbul/Turkey) and stored at +4 °C before the experiment. The pear samples were washed, peeled, sliced (2 mm thickness), and then diced into cubes of 5 mm × 5 mm. The initial moisture content of fresh pear was determined according to the standard method of AOAC No. 934.06 [25] by a vacuum dryer (EV018, Nuve, Ankara, Turkey) which was determined as 6.43 kg water/kg dry solid.

### 2.3. Biopolymer Dip-Coating Pretreatments (BDCP)

The fresh pears were subjected to five BDCPs, and uncoated pears were considered control (CO) samples. First, the samples were immersed in 0.7% *w*/*v* citric acid (as an anti-browning agent) for 5 min [26]. The coating solutions were prepared by dissolving SA, PC, XG, AG, and GE powders in distilled water to obtain 0.3% (*w*/*v*) transparent solutions using a hotplate stirrer (Wisd, Daihan Scientific. Co., Ltd., model MSH-20A, Wonju, Republic of Korea) [27]. When solutions reached room temperature, ascorbic acid (0.4% *w*/*v*) was added to each coating solution. The samples were dipped in the coating solutions for 30 min and then were taken out [10]. Only the samples coated with SA and PC separately were dipped into calcium chloride (CaCl_2_) solutions (2% *w*/*v*) to form cross-linking between the calcium with SA and PC for an extra 30 min, and then samples were taken out [17]. All experiments were replicated two times. Finally, all samples were placed on a sieve to drain the extra solution.

### 2.4. Drying Experiments

In this research, samples were dried in a laboratory-scale hot air dryer (HAD) detailed by [28] at a constant air temperature of 70 °C and air velocity of 2.0 m/s. Briefly, a ventilation fan (model No. AP-205006A5L) blew the air to the humidifier section for saturating of air at a temperature of 25 °C. The air velocity was adjusted to 2.0 m/s by a rotameter. The saturated air passed through the heater, and an ENDA ET2011 PID temperature controller adjusted the temperature (±0.1 °C). The dried air entered the drying chamber, and the weight loss data was recorded online every 1 min (software Rs weight-Ver. 5.10) by a precious balance (Fz-500i/AND, Japan) (±0.001 g). The drying operation terminated when the moisture content was less than 0.20 g water/g dry solid. The experiments were replicated two times, and average values were reported.

### 2.5. Rehydration Process

The rehydration process of dried pears was performed by dipping the dried samples in distilled water (mass of dried pears to distilled water = 1:20) at a water bath with a constant temperature of 25 ± 2 °C for 120 min [19]. The weight gain of samples was determined at 15 min intervals by draining and weighing the samples with a precision balance (Fz-500i/AND, Japan) with ±0.001 g accuracy. Subsequently, the samples were put back into the rehydration medium immediately. All experiments were conducted in duplicate [27]. The rehydration capacity of the dried samples or percentage of water gain can be calculated from the weight gain of samples during the rehydration process according to Equation (1) [23].

(1) $$Weight\;gain\left(\%\right)=\frac{W_{r}-W_{d}}{W_{d}}\times 100$$

where $W_{r}$, and $W_{d}$ represent the weights of the rehydrated and dried samples (g), respectively.

### 2.6. Theoretical Backgrounds

#### 2.6.1. Drying Kinetics

The drying rate and moisture ratio of samples were determined according to the following formulas, Equations (2) and (3), respectively [29].

(2) $$DR=\frac{M_{2}-M_{1}}{t_{2}-t_{1}}$$

(3) $$MR=\frac{\overset{-}{M}-M_{e}}{M_{0}-M_{e}}$$

where *DR*, $M_{2}$, $M_{1}$, represent the drying rate (kg water/kg dry solid min), the moisture contents (kg water/kg dry solid) at the drying time $t_{2}$ and $t_{1}$ (min), respectively. Additionally, *MR*, $\overset{-}{M}$, $M_{e}$, and $M_{0}$ are moisture ratio (dimensionless), average, equilibrium, and initial moisture contents (kg water/kg dry solid), respectively.

#### 2.6.2. Mathematical Modeling of Thin-Layer Drying Curves

A nonlinear regression analysis was used to fit the *MR* curve with the semi-theoretical Midilli Kucuk (Equation (4)) and empirical Wang Singh (Equation (5)) models by the Levenberg–Marquardt algorithm (SPSS Statistics 23, IBM, 2015). The goodness of fit was evaluated according to the statistical criteria of the *R*^2^ (coefficient of determination), *RMSE* (root-mean-square error), and *χ*^2^ (reduced Chi-square), shown in Equation (6), Equation (7), and Equation (8), respectively [29].

(4) $$MR=a\exp\left(-kt^{n}\right)+bt$$

(5) $$MR=1+at+bt^{2}$$

(6) $$R^{2}=1-\left[\frac{({\sum_{i=1}^{N}{MR}_{pre,i}-{MR}_{exp,i})}^{2}}{{(\sum_{i=1}^{N}{\overset{-}{MR}}_{pre,i}-{MR}_{exp,i})}^{2}}\right]$$

(7) $$RMSE=\sqrt{\frac{\sum_{i=1}^{N}{\left({MR}_{pre,i}-{MR}_{exp,i}\right)}^{2}}{N}}$$

(8) $$\chi^{2}=\frac{\sum_{i=1}^{N}{\left({MR}_{exp,i}-{MR}_{pre,i}\right)}^{2}}{N-n}$$

where ${MR}_{exp,i}$ and ${MR}_{pre,i}$ are the experimental and predicted moisture ratios, respectively. Additionally, *N* represents the number of observations, and *n* shows the number of model constants.

#### 2.6.3. Effective Moisture Diffusivity (D_eff_)

The diffusion coefficient of samples was calculated according to Fick’s second law of diffusion (Equation (9)) utilizing a linear regression analysis by SPSS Statistics 23, IBM, 2015.

(9) $$MR=\frac{8}{\pi^{2}}\sum_{0}^{\infty }\frac{1}{{\left(2n+1\right)}^{2}}\exp\left\{-\frac{{\left(2n+1\right)}^{2}}{4}\frac{\pi^{2}D_{eff}t}{{x_{1}}^{2}}\right\}$$

where $D_{eff}$ represents the diffusion coefficient (m/s²), *n* is a positive integer, *t* denotes the drying time (s), and $x_{1}$ shows the half-thickness of the pear samples (m).

#### 2.6.4. Momentum Transfer

Equation (10) was used to calculate the friction drag force *F_D_* (N). In the laminar boundary layer, when the Reynolds number (${Re}_{L})$ is lower than $5\times 10^{5}$ (Equation (11)), the friction drag coefficient ${(C}_{f})$ can be determined from Equation (12) [30].

(10) $$F_{D}=\frac{1}{2}C_{f}A\rho V^{2}$$

(11) $${Re}_{L}=\frac{LV\rho }{\mu }$$

(12) $$C_{f}=\frac{1.33}{{Re}_{L}^{1/2}}$$

where *L*, V, ρ, μ, and A are the characteristic length of the pears (m), the velocity of drying air (m/s), air density (kg/m³), the viscosity of drying air (kg/m·s), and surface area of pears (m^2^), respectively.

#### 2.6.5. Average Convective Heat Transfer Coefficient (H_heat_)

Equation (13) was used to evaluate $h_{heat}$ (W/m²·K) at the laminar boundary condition.

(13) $$Nu=\frac{h_{heat}L}{k_{air}}=0.664{Re}^{0.5}{Pr}^{1/3}$$

where *Nu*, *𝑘_air_*, and *Pr* are the Nusselt number, the thermal conductivity of drying air (W/m·K), and the Prandtl number, respectively.

#### 2.6.6. Average Convective Mass Transfer Coefficient (H_mass_)

A Chilton–Colburn analogy was used to calculate $h_{mass}$ (m/s) (Equation (14)) when 0.6<Pr<60 and 0.6<Sc<3000 [29].

(14) $$\frac{h_{heat}}{h_{mass}}=\rho C_{p}{\left(\frac{\alpha }{D_{AB}}\right)}^{2/3}=\rho C_{p}{Le}^{2/3}$$

where $C_{p}$, α, $D_{AB}$, Le, and Sc represent the specific heat of the air (J/kg·K), air thermal diffusivity (m²/s), and Lewis and Shmidt numbers, respectively.

#### 2.6.7. Thermophysical Properties of Pears

Thermal conductivity k (W/m·K) (Equation (15)), specific heat $C_{p}$ (J/kg·K) (Equation (16)), and density ρ(kg/m^3^) (Equation (17)) of the samples were determined using the moisture content.

(15) $$k=0.148+0.493M_{wb}$$

(16) $$C_{p}=(1.26+2.97M_{wb})\times 1000$$

(17) $$\rho =770+16.18M_{db}-295.1\times exp({-M}_{db})$$

where $M_{wb}$ and $M_{db}$ are the wet- (%) and dry- (kg water/kg dry solid) basis moisture contents, respectively [29].

#### 2.6.8. Mathematical Modeling of Rehydration Kinetics

The Peleg model was used to evaluate the rehydration kinetics of samples according to Equations (18) and (19) [19]. First, the rehydration ratio (*RR*) was determined from Equation (18), and *RR* was plotted vs. rehydration time. Then, the rehydration curves were fitted to the Peleg model (Equation (19)) via the Levenberg–Marquardt algorithm by the IBM SPSS Statistics V 23, 2015 software. After that, the suitability of the model for predicting the rehydration behavior of dried pears was determined by the *R*^2^, *RMSE*, and *χ*^2^.

(18) $$RR=\frac{W_{r}}{W_{d}}$$

(19) $$RR=1+\frac{t}{a_{1}+a_{2}t}$$

where *RR*, $W_{r}$, $W_{d}$, t are the rehydration ratio, the weight of the rehydrated sample (g), the weight of dried samples (g), and rehydration time (min), respectively. $a_{1}$ represents the rate constant (kinetic parameter) with a dimension of time (min), and $a_{2}$ denotes capacity constant (dimensionless) [19,20].

### 2.7. Quality Evaluations

#### 2.7.1. Color Measurement

A Hunter colorimeter (Minolta CR-400, Europe) was used to determine the L* (lightness), a* (redness/greenness), and b* (yellowness/blueness) color parameters to evaluate the color properties of the coated dried samples. In addition, the total color difference (Δ*E*) between the samples coated with CO was calculated by Equation (20). The experiments were replicated three times, and average values were reported.

(20) $$∆E=\sqrt[{2}]{{\left(∆L^{*}\right)}^{2}+{\left(∆a^{*}\right)}^{2}+{\left(∆b^{*}\right)}^{2}}$$

#### 2.7.2. Scanning Electron Microscopy (SEM)

The microstructure of the dried pears was observed by SEM. The cube shape (5 × 5 × 2 mm) fresh pear samples (Section 2.2) were coated with biopolymers (Section 2.3) and dried by hot air (Section 2.4). The surface of the samples should be conducive for SEM analysis; thus, the prepared samples were mounted on a stub and coated with a thin film of gold using an Emitech Sputter Coater SC7620. The setting conditions were 10 mA, 60 s using argon gas, and a vacuum of 0.1 mbar. Then, the samples were viewed in a Jeol Neoscope JCM-5000 scanning electron microscope, accelerated at 10 kV and under a high vacuum.

### 2.8. Determination of Total Polyphenols and Antioxidant Activity

#### 2.8.1. Extraction Process

The extraction was carried out according to the method described by Raupp et al. (2010) [31] with some modifications. Briefly, 0.3 g of dried pear powder (particle size < 0.5 mm) and 9 mL ethanol (80% *v*/*v*) were mixed and sonicated (200 W, 40 kHz) for 30 min at 25 °C in an ultrasonic bath (Protech Ultrasonic Bath PMYU 4-Istanbul, Turkey). Subsequently, the mixture was centrifuged (Rotofix 32 A Hettich, Andreas Hettich GmbH & Co.KG, Tuttlingen, Germany) at 4000 rpm for 25 min, and the supernatant was passed through a 0.45 µm filter. Then, it was completed with 9 mL of extraction solvent. Extraction was performed in duplicate. The prepared extracts were kept at −18 °C until the analyses.

#### 2.8.2. Determination of Total Phenolic Content (TPC)

The TPC was determined according to the method used by Singleton and Rossi [32]. Briefly, 0.9 mL of pure water and 4 mL of Folin–Ciocalteu reagent (0.2 N) were added to 100 µL of extract and vortexed. Then, 5 mL of sodium carbonate (Na_2_CO_3_) solution (75 g/L) was added, vortexed, and kept at 25 °C for 2 h in a dark place. The absorbance was measured using a UV/Vis spectrophotometer (T60 PG UV-Vis, PG Instruments Limited, Lutterworth, UK) at 765 nm. The results were expressed as mg of gallic acid equivalent (mg GAE)/100 g DM of pear samples. Measurements were preferred in duplicate.

#### 2.8.3. Determination of Total Antioxidant Activity (TAA)

The TAA was determined by DPPH (2,2-diphenylpicrylhydrazyl) method [33]. Briefly, 3.9 mL of 6 × 10^−5^ mol/L fresh DPPH solution was added to 100 µL extract and was incubated in a dark place (25 °C, 30 min). Then, the absorbance at 515 nm was measured with UV/Vis spectrophotometer (T60 PG UV-Vis, PG Instruments Limited, Lutterworth, UK). Measurements were done in duplicate, and the scavenging activity percentage was determined by Equation (21):

Scavenging activity (%) = [(Abs_control_ − Abs_sample_)/Abs_control_] × 100 (21)

### 2.9. Characterization of Dried Pear

#### 2.9.1. Crystallinity Determination (XRD)

The crystallinity of the samples was evaluated using an X-ray diffractometer (PANalytical *X’Pert* PRO, Almelo, The Netherlands) with the scanning speed, angle range, and sampling intervals of 2°/min, 10–40° (2*θ*), and 0.02°, respectively [27].

#### 2.9.2. Differential Scanning Calorimeter (DSC)

The thermal properties of the samples were determined using a differential scanning calorimeter (DSC-Q10, TA Instruments, New Castle, DE, USA) by heating from 30 to 120 °C with 10 °C/min [27].

#### 2.9.3. Fourier Transform Infrared Spectroscopy (FTIR)

The infrared spectrum of the samples was evaluated by an FTIR Spectrometer (INVENIO, Burker Corporation, Billerica, MA, USA) at the absorbance spectra of 400–4000 cm^−1^ with a resolution of 4 cm^−1^ [27].

### 2.10. Statistical Analysis

The experimental results were compared with Tukey’s tests by the analysis of variance (ANOVA) (at the 95% significance level) with the SPSS Statistics 23 program (IBM^®^ SPSS 2015, Armonk, NY, USA) and OriginPro 2021 (Origin Lab, Northampton, MA, USA).

## 3. Results and Discussion

### 3.1. Drying Kinetics

**Figure 2:**
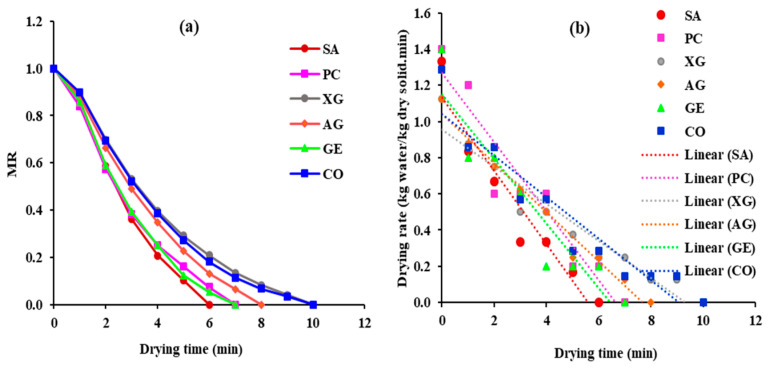
(**a**) Variation in moisture ratio with drying time; (**b**) Drying rate of samples against drying time.

The moisture ratio (*MR*) and drying rate (*DR*) are shown in Figure 2. With the extension of the drying operation, the *MR* of all samples exponentially decreases (Figure 2a). As samples are exposed to the hot air flow, the heat is transferred from the air to the surface of the samples (by convection) and then to the inside part of the samples (by conduction). Simultaneously, moisture is first transferred from the inside of the sample to the surface and then from the surface to the drying air [29]. Consequently, as drying time increases, a reduction is observed in the moisture content and *MR* of samples. As can be seen in Figure 2a, SA, PC, GE, and AG samples dry faster than the CO samples. However, the decreases in the *MR* of XG samples show similarity with the CO samples, confirming that coating with XG cannot improve the drying behavior of pears. The structural difference among coating materials creates different properties, affecting their thickening, gelling, emulsifying, and film formation properties [34].

SA and PC are linear, anionic polysaccharides, while AG is a branched polysaccharide. Furthermore, XG is linear substituted anionic polysaccharides. The fastest decreases in the *MR* belonged to the SA sample. Alginates are linear copolymer polysaccharides composed of β-(1→4)-linked D-mannuronic acid (M-block) and α-(1→4)-linked L-guluronic acid (G-block) units. The alginate, in the presence of multivalent or divalent ions usually (Ca^2+^), forms a gel-like structure [35]. The guluronic acid block creates cross-linking with calcium due to a three-dimensional network of G-block, named “egg box”, and calcium cations form the salt bridge between two adjacent polymer chains. The carboxylate groups with the negative charge in the structure of SA make it very soluble in water [34]. Therefore, the fast reduction in the *MR* of SA samples could be related to the chemical nature of SA with high polarity, high water solubility coefficient, and low moisture barrier properties. It created high permeability against water vapor as well as low resistance to mass transfer (water), causing a fast decrease in *MR* [10].

Furthermore, with the extension of the drying process, *DR* continuously decreases (Figure 2b). For all samples, a constant rate period is not observed, and drying starts from the falling rate period. Accordingly, diffusion is the dominant mechanism in controlling the drying rate. The coating pretreatment shortens the drying time by a maximum of 33.33% (SA samples), while XG slightly increases the drying time (4.76%) compared with the uncoated samples. Additionally, the drying time of PC, GE, and AG is found to be lower than the CO samples, consistent with the trend of *MR* curves displayed in Figure 2a. The high moisture content of samples at the beginning of drying creates a sufficient moisture gradient and driving force for drying; therefore, the *DR* of samples is found to be higher values at the start of drying [10]. Meanwhile, as drying progresses, lower values of *DR* are observed at the subsequent stages of drying, attributable to the gradual reduction in the water content, driving force, and moisture gradient of the sample [29]. Our results are in agreement with other studies [10,29]. This non-thermal processing significantly can be used as a green technology for improving drying kinetics and saving energy at food drying factories.

### 3.2. Mathematical Modeling of the Drying Process

The best equations for characterizing the drying behavior of products and predicting process parameters can be found using mathematical modeling, a useful tool for simulating drying processes. The drying kinetics of materials with uniform thickness (such as thin layers) can be studied using Lamped parameter models, which can be divided into theoretical, semi-theoretical, and empirical models. In this study, Fick’s second law of diffusion is used as a theoretical model (Section 3.2). The results of the mathematical modeling of the Midilli and Kucuk model (semi-theoretical) and Wang and Singh model (empirical) are detailed in Table 1. The model parameters were determined by nonlinear regression where the *R*^2^, *RMSE*, and *χ*^2^ range from 0.9440 to 0.9865, 0.03944 to 0.08292, and 0.002800 to 0.009600, respectively. The Midilli and Kucuk model with the highest *R*^2^ and lowest values of *RMSE* and *χ*^2^ could be used for the prediction of the drying behavior of all samples. Different authors reported that the Midilli and Kucuk model is suitable for showing the drying behavior of various food products such as apples [28], celery [29], and bananas [36].

### 3.3. Effective Moisture Diffusion Coefficient (D_eff_)

The *D_eff_* of the pears varies from 2.332 to 3.256 × 10^−9^ m^2^/s (Table 2), in the general range reported for foodstuff (10^−12^ to 10^−8^ m^2^/s) [29]. The *D_eff_* of the samples coated by SA, PC, and GE is significantly (*p <* 0.05) higher than the uncoated samples. While no significant (*p >* 0.05) change in the *D_eff_* is observed between AG and CO. In the case of coating with XG, the *Deff* of samples significantly (*p <* 0.05) decreases. The maximum *D_eff_* value is observed in GE samples. GE is an important fibrous protein biopolymer with a wide application in food, pharmaceutical, and many different industries [37]. Due to its characteristics, such as good gelling and foaming properties, GE is used as a coating and edible film in food industries. In addition, it has good water-holding properties [38], making it suitable for use in pretreatment coating processes. Generally, the addition of a plasticizer (such as water) to the protein-based coatings and films can improve their functional properties, such as their permeability [34]. GE is composed of α, β, and γ-chains in the form of a single chain, two covalently cross-linked α-chains, and three covalently cross-linked α-chains, respectively [39]. The interaction between the amino acids of adjacent chains by H-bonds forms the alpha-helix secondary structure [34]. In the aqueous solution, when gelatin is dissolved in hot water, it produces a semi-flexible single coil microstructure that is not aggregated [38,40], and the gelatin network traps water molecules at the sol-gel transition temperature (gelation temperature approx. 35 °C). Being cooled below this temperature, it undergoes a conformational coil-helix transition, aggregates, and attempts to partially reform the secondary structure consisting of random coils (amorphous) and triple-helixes (junction zones) [37,38,40]. At temperatures lower than 30 °C, the disordered coiled partially reforms to the helical matrix with a higher ordered structure (polymer crystallization) [38]. When GE coating is dried at room temperature, it creates ‘‘cold-dried’’ films or ‘‘helical gelatin’’ [40]. However, drying at temperatures higher than sol-gel transition temperature (>35 °C) creates coil structures, known as ‘‘coiled gelatin’’ or ‘‘hot-dried’’ films [40]. In our study, the high affinity of the GE network to trapped water from pear samples, along with drying at a temperature above the sol-gel transition temperature, changed the microstructure of the coating, significantly increasing the *Deff* values of GE compared with CO samples.

The maximum decreases in the drying time are observed in the SA samples (33.33%), followed by GE (28.57%) and PC (23.81%), meaning that the non-thermal coating pretreatments with SA, GE, and PC significantly decrease the drying time of pear, which can significantly reduce the energy consumption of dryers in an industrial scale. The AG samples showed a 14.29% decrease in drying time, while XG showed a 4.76% increase in drying time, confirming that XG is not suitable for improving the drying time of pears. Similar results about the effect of XG on the drying time and *Deff* were reported by Satorabi, Salehi, and Rasouli (2021) [41]. The authors revealed that coating of apricot slices in XG solution (0.6% *w*/*w*) before infrared drying with the IR powers of 150 and 375 W decreased the values of *D_eff_* compared with the uncoated samples. XG is a linear anionic heteropolysaccharide consisting of a linear glucose chain linked by β-(1→4) glycosidic bonds and a trisaccharide side chain. Due to the hydroxyl groups of XG, it produces hydrogen bonds in aqueous solutions [42]. XG has a high moisture retention capacity and can increase the stickiness properties of samples. Decreases in the *Deff* of XG samples could be related to the high water-holding capacity of XG, capable of increasing the stickiness of samples. As a consequence, it could restrict the diffusion or transfer of moisture in the sample matrix, causing an increase in the drying time and a decrease in the *Deff*.

### 3.4. Transport Properties

Modeling and simulation are known as useful methods for describing operational mechanisms and transfer phenomena. Drying of agricultural products consists of the transfer of heat to the food products, movement of moisture from food to its surface, and evaporation of water to drying air. A hot air dryer is known as a complex unit operation due to the simultaneous transfer of heat, mass, and momentum during the drying of food materials [28]. The transport properties such as friction drag force, convection heat, and mass transfer coefficient were affected by the external condition of the drying process, such as speed, density, viscosity, specific heat, and thermal diffusivity of drying air, as well as surface area and the thickness of samples. Convective mass transfer coefficient (*h_mass_*) and convective heat transfer coefficient (*h_heat_*) are important parameters indicating the characteristics of mass and heat transfer throughout the drying process. Generally, the *h_heat_* and *h_mass_* are determined by the dimensionless number of the Nusselt number and the Shmidt numbers, respectively. *h_mass_* can be determined by using the Chilton–Colburn analogy. Accordingly, as external conditions for all samples were the same, the calculated transfer parameters were valid for all coated and uncoated samples. *Re*, *F_D_*, *h_heat_*, and *h_mass_* are determined as 506, 6.104 × 10^−6^ N, 76.55 W/m^2^·K, 0.0636 m/s, respectively. Our results are in agreement with other studies [28,29,43]. As expected, our results show higher values of transport parameters in comparison with those of [28] in the drying of apples and in foam mat drying of mango due to the higher air velocity in the current study. Additionally, compared with the transport properties of celery root chips [29], as the characteristic length of our square samples is less than triangle shape samples of celery root, the values of friction coefficient are comparatively lower, and heat and mass transfer coefficients are higher in the current study compared with results reported by the authors.

### 3.5. Thermophysical Properties

The thermophysical properties of the samples were determined according to the moisture content of the samples (Table 3). A material’s ability to conduct heat is determined by its thermal conductivity. In addition, the amount of energy needed to raise a substance’s temperature by one degree per unit mass is known as specific heat. The highest and lowest values are related to the PC and SA samples, respectively. After PC, CO samples show high values of *k*, *Cp*, and *ρ*, likely related to the high amount of water, while coating with SA with a decrease in the moisture content of samples reduces the related parameters. Our results are in good agreement with other studies [29].

### 3.6. Rehydration

#### 3.6.1. Weight Gain

**Figure 3:**
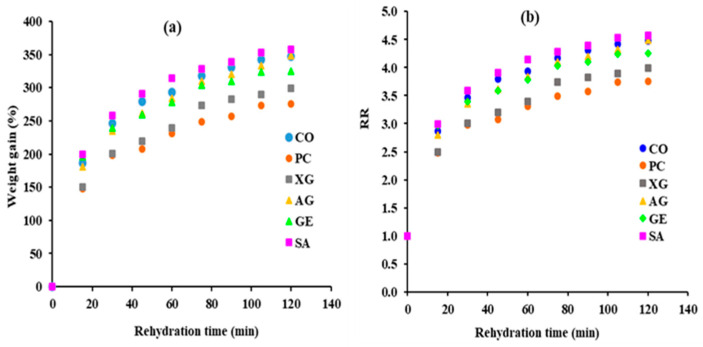
(**a**) Water uptake during the rehydration process and (**b**) rehydration ratio (RR) vs. rehydration time.

Water absorption during rehydration is a complex process, affected by various parameters such as types of food, chemical composition, microstructure, pretreatment and drying methods, as well as rehydration parameters [10]. The rehydration capacity of dried pears was evaluated according to the weight gain of samples during the rehydration process (Table 4 and Figure 3a). The amount of absorbed water increases at the start of the rehydration process and then gradually decreases (Figure 3a). The significant water gain during the first stage of rehydration could be explained by the rapid filling of pores and holes near the surface. However, as capillaries fill up with water, a reduction in water absorption is observed, indicating that the system has finally attained an equilibrium situation [23]. The maximum percentage of weight gain, 357.31%, is found in the SA samples (Table 4). While the lowest value was observed in the PC samples, in agreement with another study [27], reporting that coating turmeric with PC decreases the rehydration capacity and water absorption ability of dried samples. The decrease in the weight gain of the samples coated with XG and GE compared with CO is statistically significant (*p* < 0.05). Generally, the rehydration capacity of the dried sample is related to the degree of cellular and microstructure disruption of samples during drying. Accordingly, the rehydration of dried pears can be affected by the nature of the internal porous structure, influenced by coating materials and drying conditions [10]. Therefore, the coating of samples with PC, XG, and GE followed by drying could not protect the tissue from breakdown, resulting in hindered water absorption and slower and less rehydration capacity of dried pear. However, an increase in the weight gain of the samples coated with SA and AG is observed (Table 4), proving that the use of SA and AG could improve the rehydration capacity of dried pears. It could be explained by more preservation of the pear’s cellular structure during drying by SA and AG compared with other biopolymers, leading to an improvement in the replacement of water in dried pears during the rehydration process. Shi, Tian, Zhu, and Zhao (2019) [10] reported that the rehydration amount of dried scallop adductors coated with SA was slightly lower than that of the uncoated samples. This inconsistency with our results can be related to the difference between the samples (fish vs. fruit) and the drying method (heat pump drying and hot air drying).

#### 3.6.2. Mathematical Modeling of Rehydration Kinetics

Rehydration kinetics expressed by the rehydration ratio (*RR*) was modeled according to the Peleg model (Figure 3b and Table 5). The *RR* is higher in the initial rehydration step, while with increasing rehydration time, *it* gradually decreases and reaches the constant level. The same pattern was reported by different authors for the rehydration of kiwi [44], Rosa pimpinellifolia fruit [45], and squid fillets [24]. The Peleg model fits well with the experimental data (Table 5). The *R^2^* values range from 0.9665 to 0.9941, demonstrating that the Peleg model is acceptable for predicting the water absorption of dried pears during rehydration. In addition, the *RMES* and *χ^2^* fall within the range of 0.0414 to 0.0910 and 0.0020 to 0.0116, respectively. Among the parameters, *a*_1_ gives information about the rate of water absorption during the rehydration process at the primary step of rehydration, and it is linked to the mass transfer rate [19]. In addition, its reciprocal (*1/a*_1_) could be considered as a diffusion coefficient during rehydration. The lower *a*_1_ represents a higher initial absorption of water [24]. According to the modeling, the *a*_1_ value of SA and GE samples are about 0.884 and 0.876 times lower than that of CO samples (Table 5). While for PC, XG, and AG, it is 1.303, 1.443, and 1.126 times higher than CO samples, indicating the poor rehydration ability of these samples. The *a_2_* mainly shows the maximum capacity of the sample for absorption of water during long-term rehydration, which can be affected by the chemical composition and the physical structure of samples [19,21,24]. The $a_{2}$ values slightly varies among the samples, similar to the results reported by [20] for the rehydration kinetics of dried mango. Coating of samples with different materials can change the chemical composition of samples which may promote variation in $a_{2}$ during the dehydration process. Different authors used the Peleg model for modeling the rehydration kinetics of dried squid fillets [24] and dried mango [20]. In conclusion, the rehydration characteristics are influenced by the types, chemical composition, and microstructures of dried foods [24].

### 3.7. Quality Parameters

#### 3.7.1. Color

Coating of samples with PC, XG, AG, and GE significantly (*p* < 0.05) decreases the brightness of samples (L* value) compared with CO, except for the SA sample (Table 4). The maximum L* values of CO could be related to the porous structure of the sample, causing more reflection of light and an increase in the brightness of the dried pears [12]. The a* values of all coated samples are significantly (*p* < 0.05) lower than CO, probably related to the change in the color of biopolymer coating material during drying, which could reflex the green color in the coated products compared with CO. In general, the yellowness of coated samples displaying b* values is higher than CO. The highest b* value (yellowness) is observed in the GE sample, attributed to the natural yellow color of gelatin. The highest total color difference Δ*E* is observed in the GE, while the minimum change belongs to the AG. Different types of biopolymers affect the color properties of pear at different levels. Compared with CO samples, dried coated pears resulted in increasing L* and b* values and decreasing a* values, which indicates a decrease in the non-enzymatic browning reaction during drying [46]. Among the biopolymers, SA seems to be the best coating material to improve both the drying kinetics and color properties of pears. Our results are in agreement with other studies about the effect of coating pretreatment before drying on the color properties of celery root and apricot [29,46].

#### 3.7.2. Microstructure Evaluation

**Figure 4:**
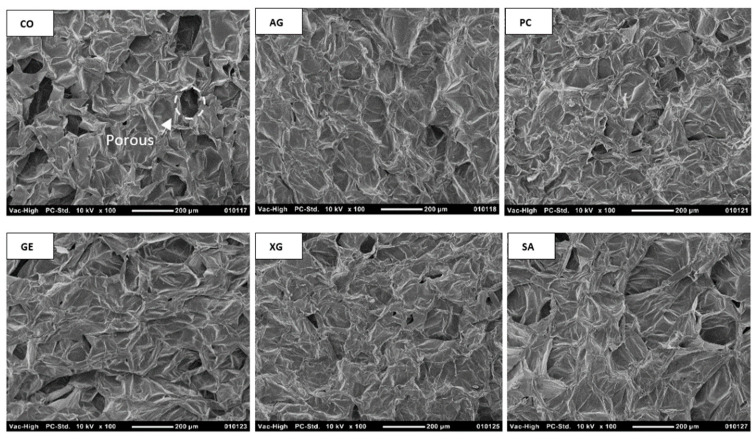
Microstructure images of pears with different biopolymer-dip-coating pretreatments.

Significant differences are observed between the surface structure of CO and the coated samples (Figure 4). The CO shows a honeycomb cell structure with large and irregular pores, a clear cell wall outline, and a homogeneous cell size. However, the porosity of the coated samples decreases due to the penetration of coating material into the cellular structure of pears [17]. An expansion in the cell size is observed in the SA samples; however, the cell wall outline is more obvious than that in other coated samples. The formation of large channels and pores due to the damage to the integrity of the cellular matrix could be the main reason for enhancing moisture diffusion during drying and water absorption during rehydration. However, a decrease in the cellular size in the PC is observed, attributable to the shrinkage of the sample during drying. Cell shrinkage during drying produces internal pressure, acting as the driving force for the migration of moisture from the inside of the sample to its surface [28]. Consequently, shrinkage with the formation of water flow (hydrodynamic flow) increases the *D_eff_* and improves the drying kinetics of PC samples. The AG coating slightly preserves the cell wall outline, improving the facility in the rehydration process of samples. Additionally, GE samples show high cell structure disruption and irregular microstructure, likely related to the drying at a temperature higher than the sol-gel transition temperature, changing the microstructure of samples. According to the damage to the integrity of the cellular matrix, the resistance against moisture diffusion decreases, and fast drying is observed in the GE samples. The XG shows a compact structure without channels. The integrity of the cell wall is destroyed, and it forms a dense layer on the surface of the pear, which could act as a barrier against water diffusion. This barrier decreases the *D_eff_* and increases the drying time of XG samples compared with CO samples. Our results are in agreement with other studies [17,27,29].

### 3.8. Total Polyphenol Content (TPC) and Total Antioxidant Activity (TAA)

TPC and the antioxidant activity of the ascorbic acid-enriched samples are detailed in Table 4. The values of TPC of the coated samples significantly (*p* < 0.05) increase compared with that of CO. The influence of biopolymers in the preservation of TPC is observed as AG > GE > SA > PC > XG > CO. However, there are no significant differences (*p* > 0.05) between the SA and GE. According to the microstructural properties of samples, biopolymer coating could create a cover on the pear surface, probably decreasing the degradation of bioactive compounds in the pear. In addition, they act as a good carrier to carry and protect the added bioactive compounds. Rodriguez, Soteras, and Campanone (2021) [18] demonstrated that coating pears before osmotic drying with SA better preserved the TPC than coating them with PC, in agreement with our study. Kayacan, Sagdic, Doymaz, and Karasu (2022) [12] reported that the TPC of Asian pears dried with a hot air dryer (2 m/s and 50 °C) was 111.59 mg GAE/100 g dry matter. However, in our study, higher values of TPC of CO samples are related to the use of higher drying temperature (70 °C), resulting in polyphenol synthesis [13]. Guiné et al. (2015) [47] reported that the TPC of the pear dried at 60 °C was 336 mg GAE/100 g dry matter, while at 70 °C, it increased to 348 mg GAE/100 g dry matter. As expected, the green technology of coating pears in different ascorbic acid-enriched solutions significantly increases the scavenging activity (Table 4). The highest values of TAA belong to the AG sample; however, the differences between AG and SA samples and between SA and GE samples are not significant (*p* > 0.05). An increase in the antioxidant activity of coated food before drying has been reported in the literature for grapefruit slices [48], raspberries [9], and turmeric [27]. Furthermore, it was stated that the TPC and TAA of pear samples dried at 60 °C and 70 °C had a linear relation [47]. 

AG is an ionic highly-branched polymer consisting of a complex combination of arabinogalactan oligosaccharides, polysaccharides, and glycoproteins [34]. It is successfully used as a coating material to preserve the quality of dried foods, such as tomato slices [44], grapefruit slices [49], and grapefruit slices [48]. In agreement with our study, the studies conducted by Morodi, Kaseke, and Fawole (2022) [9] showed that coating red raspberries with AG before oven drying at 60 °C preserved TPC content and antioxidant activity more than an uncoated sample. In another study, the Arabic gum-gelatin solution was used for coating grapefruit slices before freeze-drying [48]. Authors indicated that increases in the preservation of TPC and antioxidant activity were observed in the coated grapefruits compared with the uncoated samples. The use of all ascorbic acid-enriched coating solutions in the current study exhibited excellent antioxidant activity compared with CO. Consequently, they can be used to the industrial extent to both improve the drying kinetics of samples and improve the health benefit of dried pear in terms of TPC and TAA.

### 3.9. Characterization of Dried Pear

#### 3.9.1. X-ray Diffraction Patterns of Pear

**Figure 5:**
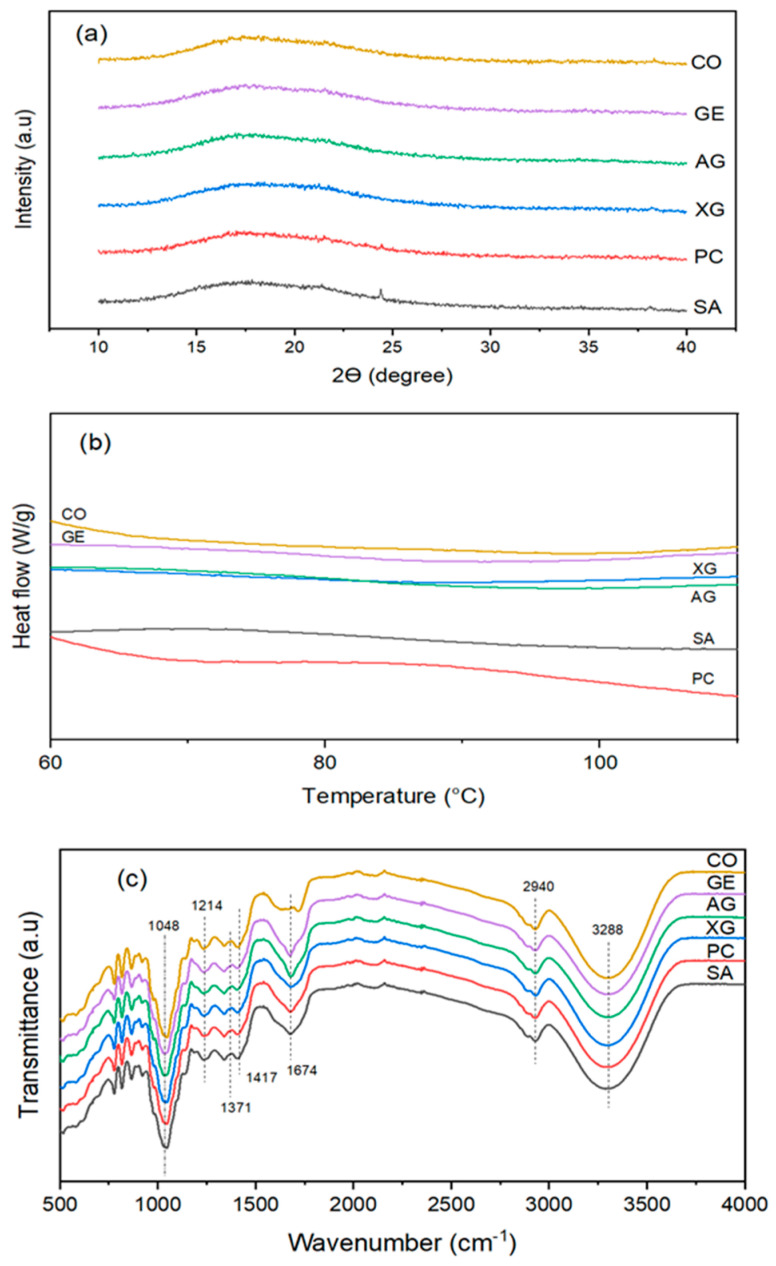
(**a**) XRD; (**b**) DSC thermograms; and (**c**) FTIR pattern of biopolymer-coated dried pears.

The results of the XRD analysis are presented in Figure 5a. The XRD diffraction patterns of all samples show minimum crystallinity at 2*θ* positions of about 15° to 20°. In other words, a quasi-amorphous microstructure is seen between the stated angles. XRD patterns showed large, broad, and undefined peaks with a lot of noise. The stability of a dried sample is greatly affected by its crystalline state. The amorphous microstructure is characterized by an irregular molecule state, and it is stated that dried powders with amorphous structures can hydrate quickly, related to the low energy levels of intermolecular bonds. Therefore, dried foods with amorphous microstructures exhibit hygroscopic properties and tend to absorb moisture more than those with crystalline structures [50]. Conversely, the absorption of moisture or dissolution of crystals in water takes place only on the outer surface of dried materials; therefore, food materials with crystalline structures show a lower level of hygroscopicity than amorphous materials [50]. In our results, all samples showed an amorphous microstructure consistent with other studies. Cano-Chauca, Stringheta, Ramos, and Cal-Vidal (2005) [51] added AG to mango juice and dried it with a spray dryer. The authors demonstrated that high molecular weight and viscosity were the important factors in the increase of the glass transition temperature (*T_g_*) of AG, favorable for an amorphous state. The alteration from amorphous to crystal situation could be observed at temperatures above *T_g_* [51]. Our results show that the pretreatment with different biopolymers does not alter the amorphous states of the pear due to the use of temperature below *T_g_*, in agreement with another study showing that the coating process had little effect on the change of crystallinity pattern of the samples. An et al. (2022) [27] demonstrated that the turmeric samples had crystalline structures, and the combination of ultrasonic and coating processes before drying did not change the crystalline structure of the samples. However, the authors revealed that a slight increase in the relative crystallinity was observed in the pretreated samples, attributed to the ultrasonic process. Ultrasound improved the reformation of crystal structure by the creation of smaller crystal regions.

#### 3.9.2. Thermal Properties (DSC)

The impacts of BDCP on the thermal properties are shown in Figure 5b. No peaks are observed in CO samples, while mild endothermic peaks (60–110 °C) are observed in the coated samples. The maximum temperature of the carbohydrate transitions is evaluated as 63.100, 62.290, 70.124, 61.249, and 62.465 °C for SA, PC, XG, AG, and GE samples, respectively. The sample shows both *T_g_* and melting temperature (*T_m_*). The peak is ascribed to the gelatinization of starch or melting of sugars contained in both pear and coating material as well as the crystal melting of starch [52]. The competition among the proteins, fibers, and starch for water absorption affects the melting temperatures of samples [27]. The mixture of sugars in the coated samples can have crystalline or amorphous microstructure. The melting temperature was dependent on the composition of carbohydrates as well as their crystalline states [52]. The breakdown of hydrogen bonds is an endothermic process, while exothermic phenomena occur due to the creation of new bonds [53]. The highest gelatinization temperature in the XG samples indicates the highest thermal gelatinization stability of these samples compared with other samples. Our drying process was carried out at 70 °C, below the *Tm* of XG. Consequently, the minimum *D_eff_* and the maximum drying time observed in the current study could be explained by the thermal properties of XG samples. Our drying temperature is not sufficient for the gelatinization and melting of XG; therefore, it produces a barrier on the pear surface against the transfer of moisture from the interior of the pears to the airflow, causing a reduction in the *D_eff_*.

Gelatinization enthalpy (Δ*H*) represents the decomposition of the crystalline region and the breakage of the double-helix structure of starch during gelatinization [27]. The *ΔH* values are observed as 1.632, 0.679, −0.148, 1.842, and 1.496 J/g for SA, PC, XG, AG, and GE samples, respectively. Negative values of Δ*H* in the XG samples are related to the un-gelatinization of XG. The higher Δ*H* confirmed that more energy was necessary to disrupt the high-order crystalline structure of starch [27]. Our results are in agreement with other studies [54]. An et al. (2022) [27] reported that coating turmeric with PC and carboxymethyl cellulose could change the thermal properties of the samples. Wang, Truong, Li, and Bhandari (2019) [54] showed that sugar profiles, binary, and the ternary mixture of sucrose, glucose, and fructose impacted the melting point and thermal properties of samples.

#### 3.9.3. FTIR Analysis

At FTIR results (Figure 5c), a broad band is observed at 3288 cm^−1^, showing an H bond. Hydrogen bonds confirm the presence of the hydroxyl group, related to the O-H stretching of phenolic compounds [55]. Additionally, the medium band at 1410 cm^−1^ indicated the O-H bending of carboxylic acids or tertiary alcohol [56,57]. The band observed at 1214 cm^−1^ is associated with the C-O stretching of phenols [57]. The band at 1674 cm^−1^ is characteristic of the aromatic rings of polyphenols and flavonoids [56]. The peak at 1317 cm^−1^ is related to the angular helix of CH_2_ in the amorphous microstructure of pear starch. In addition, the weak peak at 2940 cm^−1^ is ascribed to the C-H stretching of CH, CH_2_, and CH_3_ of carbohydrates [27]. The peaks located below 976 cm^−1^ in the FTIR pattern are associated with the C-H bonds in aromatic compounds [56]. However, a significant change in the FTIR peak and pattern is not observed after coating the samples. Therefore, the biopolymer coating of pears could not generate or destroy the organic compounds.

## 4. Conclusions

In the present study, the application of non-thermal pretreatments by biopolymer-dip-coating before the convective drying of the fresh pears was investigated. Overall, the pretreatments improved the drying and rehydration kinetics, bioactive compounds, color properties as well as microstructure of the pears. The higher affinity of SA and GE to water absorption caused a significant increase in the moisture diffusion coefficient. The maximum decreases in the drying time (33.33%) were observed in the SA samples. Compared with other coated samples, AG and SA had closer color properties to the control samples. BDCP had no clear impact on the functional groups. SA had a positive impact on the rehydration ratio and water absorption of the dried pears. This investigation provides valuable information on the green technology of biopolymer dip-coating pretreatment and demonstrates its potential to protect polyphenols and antioxidant activity in pears effectively. Significant improvement in drying characteristics by decreasing thermal processing and energy consumption would hopefully lead to the reduction of global warming and CO_2_ footprint. The application of non-thermal biopolymer dip-coating has a potential substitute for thermal pretreatments to improve both drying characteristics and quality properties of pears at the industrial scale.

## Figures and Tables

**Table 1:** Mathematical modeling of the drying process and model parameters.

Code	Models	Parameters				*R* ^2^	*RMSE*	*χ* ^2^
SA	Wang and Singh	*a* = −0.121	*b =* −0.004			0.9455	0.08281	0.009600
	Midilli and Kucuk	*a =* 0.001	*b =* −0.125	*k =* −6.703	*n* = −0.026	0.9830	0.04629	0.005000
PC	Wang and Singh	*a =* −0.146	*b =* 0.002			0.9482	0.07746	0.008000
	Midilli and Kucuk	*a =* 0.000	*b =* −0.083	*k =* −8.563	*n =* −0.034	0.9849	0. 04183	0.003500
XG	Wang and Singh	*a =* −0.127	*b =* 0.003			0.9606	0.06606	0.005333
	Midilli and Kucuk	*a =* 0.000	*b =* −0.059	*k =* −9.253	*n =* −0.029	0.9803	0.04671	0.003429
AG	Wang and Singh	*a =* −0.123	*b =* 0.001			0.9587	0.06912	0.006143
	Midilli and Kucuk	*a =* 0.001	*b =* −0.086	*k =* −7.540	*n =* −0.027	0.9865	0. 03944	0.002800
GE	Wang and Singh	*a =* −0.142	*b =* 0.001			0.9440	0.08292	0.009167
	Midilli and Kucuk	*a =* 0.000	*b =* −0.090	*k =* −8.358	*n =* −0.031	0.9796	0.05000	0.005000
CO	Wang and Singh	*a =* −0.132	*b =* 0.003			0.9561	0.07071	0.006111
	Midilli and Kucuk	*a =* 0.000	*b =* −0.058	*k =* −9.813	*n =* −0.029	0.9761	0.05222	0.004286

**Table 2:** Drying and rehydration characteristics of dried pears.

Sample	Diffusion Coefficient (*D_eff_* * 10^−9^ m^2^/s ± SD)	Change in *D_eff_* (%)	Weight Gain (%)
SA	3.072 ± 0.059 ^d^	+21.76	304.73 ± 7.05 ^b^
PC	2.858 ± 0.040 ^c^	+13.28	280.56 ± 6.34 ^a^
XG	2.332 ± 0.021 ^a^	−7.57	357.31 ± 0.82 ^c^
AG	2.609 ± 0.025 ^b^	+3.41	350.74 ± 2.91 ^c^
GE	3.256 ± 0.002 ^e^	+29.05	323.13 ± 3.34 ^b^
CO	2.523 ± 0.033 ^b^	0	343.35 ± 5.82 ^c^

* Different letters in the same column indicate differences significant at *p* < 0.05. (+): increases. (−): decreases.

**Table 3:** Thermophysical properties of dried pears.

Code	Thermal Conductivity (W/m·K)	Specific Heat (J/kg·K)	Density (kg/m^3^)
SA	0.5590 ± 0.007 ^a^	3735 ± 43.84 ^a^	851.3 ± 8.57 ^a^
PC	0.5791 ± 0.015 ^a^	3858 ± 94.75 ^a^	886.9 ± 33.76 ^a^
XG	0.5720 ± 0.012 ^a^	3814 ± 75.66 ^a^	871.3 ± 21.39 ^a^
AG	0.5687 ± 0.020 ^a^	3795 ± 121.62 ^a^	868.1 ± 32.03 ^a^
GE	0.5685 ± 0.021 ^a^	3793 ± 129.40 ^a^	868.9 ± 34.09 ^a^
CO	0.5752 ± 0.021 ^a^	3833 ± 127.98 ^a^	881.80 ± 41.42 ^a^

The letter in the same column indicate differences significant at *p* < 0.05.

**Table 4:** Color parameters, TPC, and TAA of coated dried pears.

Sample	L*	a*	b*	∆E	TPC (mg GAE/100 g DW)	TAA (DPPH % Scavenging)
SA	86.22 ± 0.31 ^d^	−4.13 ± 0.06 ^a^	21.37 ± 0.17^c^	2.42 ± 0.17 ^b^	4607.25 ± 64.08 ^d^	95.940 ± 0.31 ^cd^
PC	83.46 ± 0.16 ^a^	−3.15 ± 0.26 ^b^	18.16 ± 0.28 ^a^	3.48 ± 0.19 ^c^	3658.92 ± 116.20 ^c^	95.233 ± 0.10 ^b^
XG	85.55 ± 0.20 ^c^	−3.71 ± 0.22 ^ab^	23.38 ± 0.53 ^d^	4.01 ± 0.53 ^c^	3301.42 ± 16.65 ^b^	94.760 ± 0.17 ^b^
AG	85.42 ± 0.28 ^c^	−3.68 ± 0.22 ^ab^	20.23 ± 0.72 ^bc^	1.82 ± 0.43 ^b^	5403.92 ± 162.66 ^e^	96.440 ± 0.09 ^d^
GE	84.55 ± 0.14 ^b^	−4.01 ± 0.04 ^a^	23.95 ± 0.23 ^d^	4.92 ± 0.19 ^d^	4817.25 ± 133.91 ^d^	95.813 ± 0.18 ^c^
CO	86.47 ± 0.22 ^d^	−2.41 ± 0.31 ^a^	19.71 ± 0.83 ^b^	0 ^a^	392.25 ± 16.39 ^a^	2.350 ± 0.17 ^a^

TPC: Total phenolic content, TAA: total antioxidant activity, DPPH: 2,2-diphenylpicrylhydrazyl, SA: sodium alginate, PC: pectin, XG: xanthan gum, AG: Arabic gum, GE: gelatin; Difference lowercase letters in the same column show significant differences at *p <* 0.05.

**Table 5:** Mathematical modeling of the rehydration process of dried coated pears according to the Peleg model.

Sample	Constant Parameters		*R* ^2^	*RMES*	*χ* ^2^
SA	*a*_1_ = 4.006	*a*_2_ = 0.246	0.9941	0.041404	0.00200
PC	*a*_1_ = 5.906	*a*_2_ = 0.321	0.9665	0.080178	0.00900
XG	*a*_1_ = 6.537	*a*_2_ = 0.285	0.9705	0.089443	0.01120
AG	*a*_1_ = 5.102	*a*_2_ = 0.254	0.9756	0.087831	0.01080
GE	*a*_1_ = 3.978	*a*_2_ = 0.279	0.9603	0.091026	0.01160
CO	*a*_1_ = 4.531	*a*_2_ = 0.253	0.9905	0.053452	0.00400

## Data Availability

All data included in this study are available upon request by contacting the corresponding author.
